# Androgens and the developing hippocampus

**DOI:** 10.1186/s13293-020-00307-6

**Published:** 2020-06-01

**Authors:** Katherine E. Kight, Margaret M. McCarthy

**Affiliations:** 1 Department of Pharmacology, University of Maryland School of Medicine, 685 W Baltimore Street, Baltimore, MD 21201 USA; 2 Program in Neuroscience, University of Maryland School of Medicine, Baltimore, MD 21201 USA

**Keywords:** Estrogens, Testosterone, Spatial learning, Neurogenesis, Synaptogenesis

## Abstract

The hippocampus is central to spatial learning and stress responsiveness, both of which differ in form and function in males versus females, yet precisely how the hippocampus contributes to these sex differences is largely unknown. In reproductively mature individuals, sex differences in the steroid hormone milieu undergirds many sex differences in hippocampal-related endpoints. However, there is also evidence for developmental programming of adult hippocampal function, with a central role for androgens as well as their aromatized byproduct, estrogens. These include sex differences in cell genesis, synapse formation, dendritic arborization, and excitatory/inhibitory balance. Enduring effects of steroid hormone modulation occur during two developmental epochs, the first being the classic perinatal critical period of sexual differentiation of the brain and the other being adolescence and the associated hormonal changes of puberty. The cellular mechanisms by which steroid hormones enduringly modify hippocampal form and function are poorly understood, but we here review what is known and highlight where attention should be focused.

The brain is a hormone-responsive organ central to the production of steroids by both the gonads and the adrenals. As part of the hypothalamic-pituitary-gonadal/adrenal axes, it serves as the site of both stimulatory signals and negative feedback. Both the reproductive and stress axis differ in males and females, albeit to varying degrees, with reproduction being truly dimorphic, while stress responsiveness is context- and lifestage-dependent. The role of the brain in reproductive physiology and behavior has been intensely investigated for over five decades. Early in the process was the establishment of a central dogma, the Organizational/Activational Hypothesis of Sexual Differentiation of the Brain. This tenet is the simple idea that developmental exposure to gonadal steroids, specifically androgens derived from the fetal testis, organizes the brain along a masculine phenotype which is then activated by testicular androgens post-puberty. The organization of the feminine phenotype occurs in the absence of exposure to high levels of androgens during development, and the brain is then activated by ovarian estrogen and progestins in adulthood. The result is that adult males continuously produce large numbers of gametes and seek females with which to mate, while females cyclically mature a select few gametes and only mate in coordination with that event (with humans being a notable exception with regard to the latter). If there is a mismatch between the steroid hormone milieu of the organizational and activational periods, neither scenario is achieved. In other words, an adult female given androgens does not respond with male-typic physiology or behavior and vice versa. However, if a newborn female is treated with androgens during the critical organizational phase, and given androgens again as an adult during the activational phase, she does respond with male typic behavior, albeit without the ability to actually produce sperm. This demonstrates the capacity of the brain to be sexually differentiated independently of the body and establishes the primacy of androgen exposure during early development as a critical driver of this process.

The neural control of reproductive physiology and behavior is largely localized to key nuclei of the preoptic area and hypothalamus and thus these regions were the primary focus of interest regarding the actions of steroid hormones in the brain. Reports in the 1990s that hippocampal synaptic form and function varied across the estrous cycle of female rats [1–3] stirred great interest in the notion that non-reproduction associated brain regions and behaviors could also be modulated by hormones. However, for the most part, the emphasis was and continues to be on activational effects of steroids, with only a modest attention given to potential organizational effects.

## Modulation of hippocampal function in relation to sex and hormones

The hippocampus is a discrete telencephalic structure located in the medial temporal lobe and is required for episodic and spatial memory [4, 5] and context-dependent learning [6–8]. It is also a crucial brain region for regulation of the stress response [9–11]. Two main divisions of the hippocampal formation are recognized: Ammon’s horn, which is further subdivided into areas CA1 through CA4, and the dentate gyrus, which is a major site of neurogenesis in the adult brain. In general, two broad functional divisions are assigned to the hippocampal formation along its longitudinal axis, based on neuroanatomical connectivity, results from ablation studies, and molecular profiling. The dorsal or septal half of the hippocampus is involved in processing of spatial information and memory, and sends output projections to the anterior cingulate cortex, mammillary nuclei, and anterior thalamus. The ventral or temporal portion of the hippocampal formation, which is highly connected with the olfactory system, prefrontal cortex, amygdala, and hypothalamus, preferentially mediates stress responding [12–16]. Changes in memory, contextual learning, and stress responding are seen throughout the lifespan and are associated with changes in circulating testosterone and estradiol. Numerous cellular and molecular differences between males and females in relation to gonadal steroids have been described in the hippocampus using rodent models, and the effects of adult steroid hormones on hippocampal-dependent behaviors and the associated cytoarchitectural changes within this region of the brain, both in health and pathological dysfunction, are thoroughly reviewed elsewhere [17–21]. While steroid hormones clearly have numerous modulatory roles in the adult hippocampus, it is not generally considered a sexually differentiated region of the brain, as morphological and functional differences are small and often the consequence of the different hormonal milieu of adult males and females [22]. There is evidence, however, that the hippocampus is organized differently in males and females during development in ways that result in persistent changes in function in the face of environmental and physiological perturbations.

Hippocampal dysfunction is associated with many disorders that differ between the sexes in terms of prevalence and/or presentation, including epilepsy, schizophrenia, autism spectrum disorders, depression, and anxiety (see [23–26] and references therein). Several of these disorders are associated with prenatal and childhood risk factors which are also correlated with altered hippocampal structure. For example, childhood stress and trauma, well-established risk factors for depression and anxiety later in life, are associated with decreased hippocampal volume in adults [27–29]. Increased hippocampal volume is seen in adolescents with anxiety and post-traumatic stress disorder who have experienced early life trauma [30], and smaller hippocampal volume is seen in schizophrenia patients with a history of obstetric complications, compared to uncomplicated births [31, 32]. Numerous animal studies have revealed molecular and cellular effects of developmental influences on the hippocampus which manifest in sex-specific ways and persist into adulthood. These include altered neurogenesis in response to gestational stress or maternal separation [33–37], altered expression of neuroendocrine markers associated with anxiety-like behavior [38–40]; differences in the density of dendritic spine synapses following early life stress [41–43], and alterations in interneuron subtypes which are associated with electrophysiological changes and anxiety-like behavior in adults [44]. The wealth of data linking the hippocampus to sex-specific brain function that is programmed in early life indicates that the period of brain sexual differentiation, which is driven by testosterone and its metabolites, has important influences over the hippocampus.

## Hippocampal development in a steroid-rich environment.

The human hippocampus begins forming from the second trimester, and by 18 to 20 weeks gestation largely resembles that of the adult in overall form, with Ammon’s horn and the dentate gyrus structurally assembled, although the dorsal and ventral portions of the hippocampus continue to differentiate well into childhood [45–47]. In non-human primates and rodents, detailed cellular birthdating studies also demonstrate a protracted period of embryonic and postnatal development of the hippocampal formation involving distinct waves of cellular proliferation and migration [48–52]. In rats, Ammon’s horn is largely formed by birth, after cells that populate this subregion of the hippocampal formation have migrated from a germinal center in the medial region of the telencephalon beginning around embryonic day 16. The dentate gyrus, however, arises from a distinct germinal zone during the perinatal period and continues to form well into the second week of life. Proliferating neuroblasts migrate tangentially and radially over several days to form a third germinal zone which will give rise to the dentate gyrus. Cellular proliferation peaks here during the first postnatal week, and gradually declines until approximately 1 month of age, when it is confined to a single layer of proliferating cells in the subgranular zone, which remains a major site of neurogenesis in adults.

The protracted development of the hippocampal formation during the late gestational and early postnatal period means that the cytoarchitectural patterning is laid out during the critical period of brain sexual differentiation, and thus potentially subject to the influence of gonadal steroid hormones. Although considerably less well-characterized than in the adult hippocampus, all the components needed for both androgen and estrogen signaling are present during this early developmental period. In rodents, androgen receptor expression is found in the hippocampus as early as embryonic day 15 [53], and rises with postnatal development [54]. Nuclear estrogen receptor alpha is expressed in the pyramidal cell layers of CA1 and CA3 during the first postnatal week, after which levels decline [55–57]. Estrogen receptor beta is similarly abundant in CA1 and CA3 subregions during the first postnatal week [58], and is also present at extranuclear sites in granule cells and glia of the dentate gyrus [59]. As in other regions of the developing brain, synthesis of estradiol from circulating perinatal testosterone occurs here. Aromatase activity is evident in hippocampal tissue during the first postnatal week, declining significantly thereafter [60, 61]. The level of hippocampal aromatase activity is markedly lower than in other highly sexually dimorphic brain regions such as the preoptic area, mediobasal hypothalamus and amygdala, but nevertheless corresponds precisely with levels of estradiol content, which are highest just prior to birth and decline ten-fold over the first postnatal week [62, 63]. Interestingly, however, there are no sex differences in hippocampal estradiol content during the perinatal period or at any point measured to date across the lifespan, and the same is true for testosterone and DHT [63]. Central blockade of aromatase activity during the first day of life reduces estradiol content in the hippocampus of females, indicating that there may be a sex difference in neurosteroid synthesis during the perinatal period that functions to equalize androgen and estradiol content between males and females in this region of the developing brain [62]. There is also no concrete evidence of sex differences in estrogen and androgen receptor expression in hippocampal neurons during development, suggesting that other mechanisms may program sex differences in hippocampal function. Potential mechanisms that occur downstream of, or peripheral to perinatal steroid signaling in the brain and are important mediators of sexual differentiation include genetic and epigenetic mechanisms, differences in neuroimmune modulators, and sex-specific impacts of the environment [64, 65]. Below we discuss the evidence that androgens, either directly or via aromatization to estradiol, have both organizational and modulatory roles in the developing hippocampus, and highlight what is known about potential mediators of these testosterone-driven effects.

## Organizational effects of testosterone and its metabolites in the neonatal hippocampus

### Programming of hippocampal function

Sex differences in hippocampal-dependent learning involving spatial navigation or object memory have long been observed, although the direction and magnitude of differences in performance is influenced by numerous intrinsic and environmental factors, including the task involved, species and strain, hormonal status, rearing and housing conditions, and season (see [66] for an excellent review on the topic). The most consistent finding among rodents is an advantage for young adult males, compared to females, in spatial navigation in the Morris water maze and radial arm maze, and this finding extends to humans when tested in a virtual rendering of these tasks. Although performance in these tasks is modulated by levels of gonadal steroids in adults of both sexes, in rodents performance is also programmed developmentally by perinatal testosterone, prior to the first 10 days of life. Castration of male rats in the first two postnatal days results in poorer acquisition of spatial learning accuracy as adults, an effect which is prevented by neonatal administration of testosterone in gonadectomized animals [67–69], while neonatal administration of testosterone to females masculinizes adult spatial memory performance [67, 70, 71]. Estradiol administration to neonatal females also masculinizes performance in the radial arm maze [68, 69], indicating that the aromatization of perinatal testosterone is required for organizing spatial learning. However, in a study from Isgor and Sengelaub [72], when the androgen receptor antagonist Flutamide is administered to pregnant dams from embryonic day 16 onward, male offspring exhibit female-like spatial learning as adults, suggesting that direct effects of androgen signaling may be required, either in addition to estradiol or as a distinct mechanism prenatally. This is supported by deficient spatial learning abilities in androgen-insensitive rodent models. Male rats and mice of the testicular-feminization mutant (*Tfm*) strain, which lack a functional androgen receptor, fail to develop secondary sex characteristics due to a mutation in the androgen receptor. Levels of circulating testosterone in *Tfm* male mice are low, while in *Tfm* rats, circulating testosterone is within normal range [73], and yet in both species, *Tfm* males exhibit poorer performance in the Morris water maze than wild-type siblings [74, 75].

Although the discernment of bona fide sexual differentiation in hippocampal-dependent behaviors can be obscured by small and inconsistent differences, as well as hormonal modulation in adults, one aspect of spatial learning that is clearly organized by the perinatal surge in testosterone is the difference in navigation strategies used by males and females. Prepubertal and adult male rats use a striatum-dependent, proprioceptive approach to spatial learning in both the radial arm and Morris water maze, using their position within the geometry of the room to navigate, while adult females use a hippocampal-dependent, context-based approach to navigate [68, 76, 77]. The context-based strategy in females is activated by pubertal hormones, as preadolescent females will navigate using either proprioceptive or context-dependent strategies. When newborn males are castration-deprived of steroids, they preferentially use a context-based navigation strategy as adults [68]. Conversely, newborn females treated with testosterone or estradiol will use a proprioceptive-based strategy as adolescents and adults, in spite of the presence of the female-typic hormonal milieu [68, 76]. Together, these studies demonstrate that engagement of the hippocampus during spatial navigation is both sexually differentiated and organized by the aromatization of testosterone during early development. Mechanistically, this may occur through programmed changes in cholinergic release into the hippocampus. Greater muscarinic receptor binding is found in the hippocampus compared to the striatum in animals using a context-dependent navigation strategy [76], and sex differences in adult hippocampal acetylcholinesterase content correlate with a preference for context-dependent navigational strategies and are altered with either testosterone or estradiol treatment in neonates [78].

### Changes in hippocampal cytoarchitecture

Functional programming of spatial learning in response to perinatal steroids is associated with alterations in cellular morphology within the developing hippocampus that persist into adolescence. Pre- and postnatal testosterone exposure promotes a larger field size in CA1 and CA3 regions in males, compared to females, driven by increased dendritic arborization. This appears to be an androgen-specific effect, as dendritic arborization is promoted in females by neonatal administration of DHT, but not estradiol, and is blocked by androgen receptor antagonism in neonatal males [67, 72, 79, 80]. The density of dendritic spine synapses on pyramidal neurons in these regions is also promoted by perinatal androgen receptor signaling, which remains in juvenile animals [79].

**Fig. 1:**
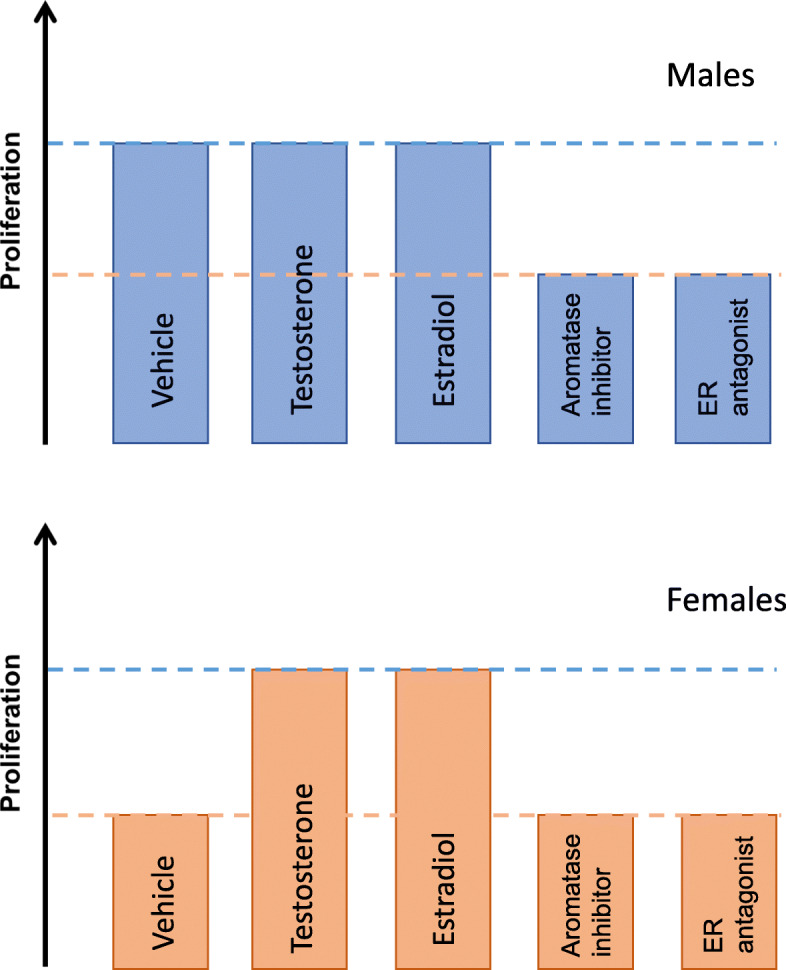
Cell proliferation in the early developing hippocampus is modulated by estradiol signaling in a sex-specific manner. Neonatal male rats have twice as many proliferating cells as females in the dentate gyrus and CA1 regions. Inhibition of estradiol synthesis or estrogen receptor (ER) antagonism decreases proliferation in males to the same levels as females, but neither testosterone nor estradiol will further increase proliferation in males above baseline. Both testosterone and estradiol increase the number of proliferating cells in females to the same level as males, while inhibition of estradiol synthesis and ER antagonism has no effect

In addition to dendritic arborization and spinogenesis, perinatal testosterone exposure promotes persistent alterations in cell type and number in the hippocampus. In adolescent rats, sex differences in astrocyte numbers in CA1 and CA3 subregions are promoted by testosterone and blocked by androgen receptor antagonism in neonates [81]. Neonatal testosterone also promotes a sex difference in cell genesis in the CA1 and dentate gyrus subregions, which is perhaps the most robust sex difference in the developing hippocampus. Male rodents have approximately twice as many proliferating cells as females during the first postnatal week [82–84], although in mice this appears to be strain-dependent (Wimer and Wimer ibid). Dividing cells can be permanently marked with the nucleoside analogue BrdU, enabling quantification of cell proliferation as well as assessment of cell survival. Administration of DHT to neonatal females increases the number of BrdU-labeled cells in neonatal females, and in males that lack a functional androgen receptor, the amount of cells thus labeled declines over a 3-week period, indicating that androgen signaling promotes cell survival in the developing hippocampus [84, 85], an effect seen in adults as well [86]. Estradiol signaling also has an important role in modulating cell genesis during development, and does so in a sex-dependent manner (Fig. 1). Inhibition of aromatase or antagonism of estrogen receptors decreases cell proliferation in neonatal males but has no effect in females and conversely, estradiol administration in neonatal females masculinizes proliferation but does not alter cell genesis in males [82, 84]. Nearly 80% of the proliferating population will differentiate into neurons in males and estradiol-treated females, whereas in untreated females, only 40% of the newly born cells become neurons. The effects of estradiol in promoting proliferation and neuronal differentiation, as well as estrogen receptor expression, have been demonstrated in mixed-sex cultures of isolated embryonic neural stem cells from rodents [87–89], although these effects depend on the dose of estradiol and presence of growth factors (see [90] for review). Thus, aromatization of testosterone modulates neonatal cell genesis in the hippocampus, likely through direct steroid receptor activation on neuronal precursors, and this action is constrained within a tightly controlled range the limits of which are expressed as a ceiling in males and a floor in females. Given that intra-hippocampal steroid content is the same in males and females, how this sexually differentiated response is achieved is unclear, although a study by Bramble and colleagues indicates that this may be determined by genetic sex. In cultured neural stem cells isolated from embryonic mice prior to the perinatal testosterone surge, a robust sex difference in RNA transcripts is found, and addition of testosterone to these cultures induces sex-differentiated alterations in the transcriptome profile. Interestingly, a majority of transcripts exhibiting baseline sex differences in embryonic neural stem cells encode factors which regulate the cell cycle [87]. Thus, the sex difference in cell proliferation in the neonatal hippocampus may be determined by a differential response to steroid hormone exposure that is programmed by the sex chromosomes.

The number of proliferating cells in the developing hippocampus may also be regulated by microglia. These neuroimmune cells function as inflammatory mediators in the brain, responding to trauma and pathogens, but also play critical roles in normal developmental processes. During the period of brain sexual differentiation, phagocytic microglia which engulf proliferating progenitors in the developing hippocampus are more numerous in females, and this sex difference is eliminated with exogenous estradiol, in parallel with an increase in the number of proliferating cells [91]. The effects of androgen signaling on this process in the developing hippocampus are not yet known. However, in the neonatal amygdala, androgens, via upregulation of endocannabinoids, increase microglial phagocytosis of proliferating cells in males, and this programs sexually differentiated play behavior in juveniles [92]. Microglia also play a critical role in developmental synaptic pruning [93, 94], and alterations in this activity during early life have long-term consequences for the hippocampus. Knockout mouse models deficient in microglia or in which microglia phagocytosis is disrupted demonstrate decreased synaptic pruning and more immature dendritic spines in CA1 of the developing hippocampus, resulting in delayed electrophysiological maturation [93, 95], altered seizure susceptibility [93], altered circuit connectivity, and ASD-like behaviors [95]. In mice, a female-biased sex difference in the phagocytic capacity of microglia during the second postnatal week corresponds to greater synaptic spine density in the hippocampus [96]. In addition, microglia isolated from adult females have a less reactive phenotype which is maintained even when implanted into male brains. When females are given a masculinizing dose of estradiol during the first postnatal week, their microglia as adults have a male-like gene expression pattern [97], indicating that intrinsic sex differences in microglia are programmed by developmental steroid hormone exposure. Thus, perinatal testosterone may sculpt the circuitry of the developing hippocampus, either directly or subsequent to aromatization, through modulation of microglia activity.

### Cellular mechanisms

In the developing brain, cell proliferation and maturation, dendritic arborization and spinogenesis are all regulated by neurotransmitter activity [98], which in turn is modulated by steroid hormone signaling. A striking sex difference found in the neonatal hippocampus of rats is the response to the neurotransmitter GABA, which is largely depolarizing at birth and gradually shifts to a hyperpolarizing response over the first 6 days of life in both males and females [99, 100]. GABA_A_ receptor activation increases phosphorylation of the transcription factor CREB in neonatal males, but decreases CREB activation in neonatal females [101], and this sexually divergent response is likely due to differences in calcium influx. In mature neurons, GABA is the dominant inhibitory neurotransmitter and maintains a hyperpolarized membrane potential as a consequence of influx of negatively charged chloride ions through the GABA_A_ receptor ionophore. In immature neurons, however, intracellular chloride is sufficiently high that it effluxes upon receptor opening, and this depolarizes the membrane and leads to the opening of voltage-gated calcium channels, allowing calcium to flow into the cell. Calcium influx in response to GABA_A_ receptor activation in hippocampal neurons isolated from newborn females exhibits a rapid decay which is attenuated with repeated exposure to GABA receptor agonism. In males, a longer decay in the response to GABA receptor activation is seen and receptor desensitization does not occur, resulting in excitotoxic cell death. Female hippocampal neurons are masculinized in terms of the timecourse and desensitization of GABA receptor activation with systemic administration of DHT neonatally, and this correlates with an increase in the γ2 subunit of the GABA receptor [102]. The depolarizing response to GABA in hippocampal neurons from neonatal females is also prolonged with estradiol treatment, in cultured neurons from neonatal rats [103] and primates [104]. A primary mechanism of estradiol-induced enhancement of depolarizing GABA is upregulation of SPAK kinase, which phosphorylates NKCC1, the key chloride transporter promoting the inward-rectifying chloride gradient and the depolarizing response to GABA [105]. In addition to the differentiated neuronal response to depolarizing GABA between males and females, there is a sex difference in the developmental timecourse when GABA receptor activation shifts from depolarizing to hyperpolarizing. In the developing hippocampus, this occurs earlier in females and is marked by downregulation of NKCC1 expression and upregulation of the KCC2 chloride transporter, which pumps chloride out of the cell, promoting an ion gradient that results in a hyperpolarizing response to GABA receptor gating. Expression of these two chloride transporters are regulated by steroid hormones. In the developing substantia nigra, androgens downregulate KCC2 expression in both sexes, while estradiol promotes KCC2 expression in males only [106], although in the hippocampus of ovariectomized adults, estradiol promotes gene expression of NKCC1, but not KCC2 [107]. The decline in nuclear estrogen receptor alpha after the first week in the developing hippocampus may contribute to the developmental shift from a depolarizing to a hyperpolarizing response to GABA by regulating expression of these transporters and thus shifting the cellular chloride gradient.

**Fig. 2:**
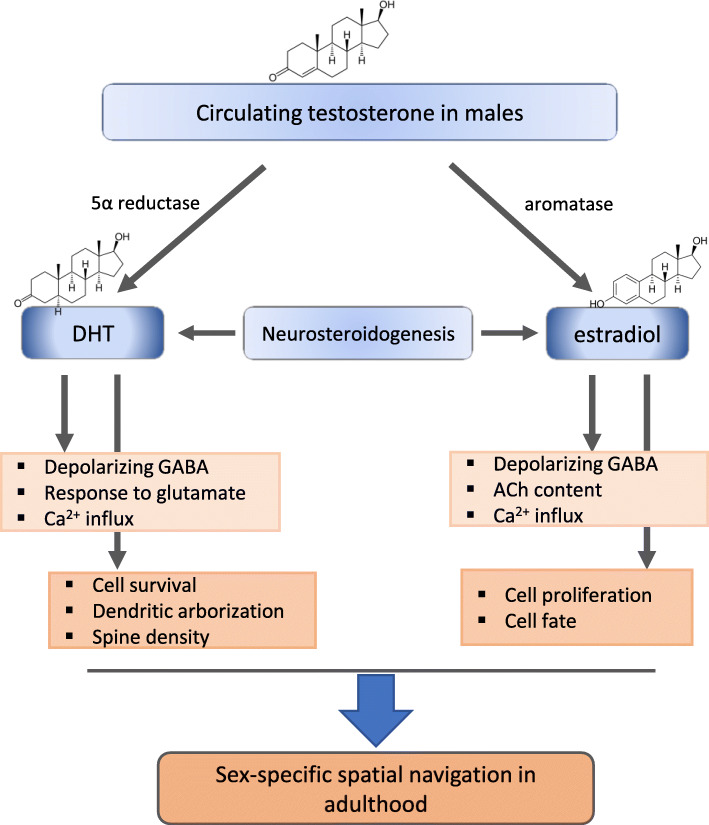
Schematic summary of the known effects of androgens and estrogens in the developing hippocampus during the critical period of sexual differentiation in rodents. The perinatal rise in circulating fetal testosterone in males provides substrate for the synthesis of dihydroxytestosterone (DHT) and estradiol in neural cells through the activity of 5-alpha reductase and aromatase, respectively. Neurosteroids are also synthesized de novo in the developing hippocampus of males and females. Androgen-specific and estradiol-specific effects of steroid hormones are found in the developing hippocampus which are most proximally represented by modulation of the cellular response to neurotransmitters. More persistent changes in the cytoarchitecture of the hippocampus are also programmed by neonatal steroids, including alterations in cell genesis, neuronal maturation, and spine synapse density. Ultimately these changes are associated with differences in hippocampal function in adults

Changes in calcium associated with neurotransmitter signaling are also modulated by the effects of neonatal androgens on the expression of intracellular calcium transporters, although the direction of these effects is not clear. DHT increases calcium influx in response to glutamate in cultured hippocampal neurons from newborn males, but not females, although changes in SERCA2 expression are elevated by DHT similarly in both sexes [108]. Androgen signaling also increases SERCA2 expression in mixed-sex cultures derived from embryonic rats prior to the prenatal testosterone surge, but decreases the response to glutamate [109], suggesting that perinatal testosterone may set up a sexually differentiated response to glutamate signaling in the early developing hippocampus. Overall, the perinatal surge in testosterone has downstream effects on cellular physiology in the developing hippocampus that result in persistent changes in cell number and morphology and program adult hippocampal function (Fig. 2).

### Epigenetic changes

The enduring nature of the cellular changes in the hippocampus programmed by a very brief developmental window of hormone exposure indicate that perinatal testosterone exposure may result in epigenetic changes, as has been shown in other, sexually differentiated regions of the brain [110]. It is increasingly clear that interactions among genetic sex, hormone exposure, and environmental factors determine the epigenetic profile of a particular brain region [111, 112], and much interest has been devoted to unraveling how these interactions contribute to normal behavior and brain disorders across the lifespan. In the context of the developing hippocampus, sex-specific alterations in DNA or chromatin modifications result from early environmental perturbations and are associated with sexually differentiated changes in hippocampal function later in life. For example, rodent models of gestational stress or early life maltreatment demonstrate increased global DNA methylation in the hippocampus of males, but not females, that persists into adolescence and is associated with greater anxiety and depressive-like behaviors [113, 114]. Perinatal lead exposure also alters DNA methylation and histone acetylation patterns in the developing hippocampus in a sex-dependent manner, and these changes are associated with increased anxiety-like behavior and poorer performance in memory tasks in males [115, 116]. Although much is known about changes in DNA methylation and histone modifications in the hippocampus throughout development [117], few studies address epigenetic modifications in this region of the brain in relation to sex or steroid hormone signaling. Tsai and colleagues [118] found a sex difference in histone acetylation in hippocampal tissue of neonatal mice which disappears when pups are treated gestationally with testosterone. Testosterone similarly induces sex-specific changes in histone acetylation in isolated embryonic neural stem cells from mice, and transcriptional changes in these cells in response to testosterone are enriched for factors involved in chromatin organization and assembly [87]. DNA methylation is also altered by exogenous testosterone in embryonic neural stem cells, where it is reduced globally in both XX and XY cells. Changes in DNA methylation in response to perinatal steroid hormones are critical for programming other sexually differentiated brain regions, as shown in the preoptic area and striatum of mice in response to testosterone [119], and in the preoptic area of rats in response to estradiol [120]. Perinatal testosterone, either directly or through aromatization to estradiol, may similarly program enduring sex differences in the hippocampus through altered DNA methylation. This may also promote the closing of a sensitive window for estradiol action in the hippocampus, as the steep decline in estrogen receptor alpha gene expression in the CA1 region after the first postnatal week corresponds with increased methylation in the promoter region of this gene [121].

### Implications for clinical outcomes

Several of the steroid-hormone-dependent processes described above have particular relevance for sex differences in the outcome of early life events that can have significant negative long-term outcomes, such as seen in neonatal seizures or hypoxia/ischemia. Clinical and animal studies have shown that neonatal hypoxia/ischemia in particular results in greater damage to the cortex and hippocampus in males, and poorer long-term outcomes compared to females with comparable damage [24, 122]. Likely mechanisms for this are the more reactive profile of microglia and elevated inflammatory cytokines in neonatal males that are present several days after hypoxia/ischemia when compared to females [123], and greater mitochondria dysfunction which precedes cell death [124, 125]. In addition, increased membrane depolarization and calcium influx in response to excitatory GABA in males results in greater excitotoxic damage, and as mentioned above, this is promoted by androgens [102]. The few studies that have tested the effects of androgens on hypoxia/ischemia outcome have shown increased infarct volume and poorer behavioral outcomes when neonates are treated with exogenous testosterone [126]. Far more attention has been paid to the effects of estradiol on neonatal hypoxia/ischemia, in large part due to the wealth of data suggesting that estradiol is neuroprotective in the context of adult stroke [127]. In the neonatal brain, animal studies demonstrate that estradiol generally mitigates hippocampal damage resulting from hypoxia/ischemia and improves outcomes later in life [128–131]; although deleterious effects on excitotoxic cell death have also been seen [132].

## Effects of pubertal testosterone on the developing hippocampus

The second developmental period in which steroid hormones can exert an organizing effect on the brain is during puberty, defined as a period of adolescence marked by a steep rise in circulating gonadal steroids. The most important outcome of puberty is the activation of sexually differentiated neural substrates by gonadal steroids in order to promote reproductive behavior. However, circuits mediating social and cognitive behaviors are also sculpted in a sexually differentiated manner during this time via a variety of cellular mechanisms (see [133, 134], for review). Evidence that hippocampal function and neurophysiology are both modulated and programmed by pubertal androgens is seen in animal and human studies.

Longitudinal imaging studies in children generally find no prepubertal sex differences in hippocampal size or morphology, but greater hippocampal volume in boys throughout puberty and adolescence, when corrected for total intracranial volume [135–140]. In terms of the relative growth trajectories between males and females, however, the data seemingly conflict. While some studies demonstrate parallel increases in hippocampal volume throughout puberty in both boys and girls [138, 141], others show sexually differentiated growth trajectories, where hippocampal volume increases steadily with advancing age and puberty in females, but growth slows in males during late puberty [137, 139, 142, 143]. This may indicate a sexually differentiated response to gonadal steroids in the hippocampus, or it may indicate a biphasic effect of androgens, where rising levels promote a positive growth trajectory, but high levels, as found in the circulation of late-pubertal males, have a negative impact on growth. This is supported by data from Wierenga et al. [140], which correlates high circulating testosterone in adolescent females with slower hippocampal growth. In addition, while the trajectory of hippocampal growth is predicted by advancing puberty status in males, in adolescent females hippocampal volume is not strongly associated with pubertal status, but instead is predicted by age [144] or circulating testosterone levels [139, 140, 142, 145]. Further support for a direct role of androgens in determining the sex difference in hippocampal volume during adolescence is found during adrenarche, which is characterized by elevated adrenal-derived androgens and where higher circulating testosterone is associated with larger hippocampal volume in girls [145].

A similar pattern of testosterone effects during puberty is seen functionally. In adolescents, response to fearful faces or performance on context-dependent memory tasks are positively associated with hippocampal activation. Test performance and hippocampal activation are both predicted by stage of pubertal development in boys and girls; however, in girls, the predictive power of pubertal stage is due almost entirely to levels of circulating testosterone, and not age or stage of puberty [146, 147]. In males, activation of the hippocampus during emotional processing is significantly greater in young boys who have familial male-precocious puberty, compared to unaffected boys of the same age [148].

Perhaps the most compelling evidence for the role of pubertal androgens in hippocampal maturation are imaging studies in adolescents with Klinefelter syndrome. Aneuploid boys with more than one X chromosome initiate puberty but are androgen deficient [149, 150] and have smaller hippocampal grey matter volume than typically developing boys [151, 152]. Treatment with a testosterone analog during adolescence normalizes hippocampal volume in Klinefelter patients [153], indicating that testicular steroids, and not sex chromosome complement, is a primary driver of hippocampal maturation during adolescence. In spite of the impossibility of direct functional experiments in humans, what emerges is that androgens have a primary role in directing adolescent maturation of the hippocampus, and the level of circulating testosterone is an important setpoint for sexual differentiation of this brain region during puberty in both sexes. Although a similar role for ovarian steroids during puberty is not supported by the imaging data in humans, a role for brain-derived estradiol in the adolescent brain, similar to the perinatal period in rodents, cannot be ruled out [154]. Peripheral blood cells from pre- and postpubertal adolescents demonstrate female-specific methylation patterns that arise during puberty for genes involved in androgen signaling and that are proximal to estrogen response elements [155], indicating that the rise of ovarian steroids during puberty may epigenetically program the response to androgens in girls.

There are relatively few studies that shed light on potential cellular mechanisms mediating the effects of pubertal testosterone on hippocampal development. Data from rhesus macaques demonstrate a role for adolescent testosterone in regulating cell survival and differentiation in the hippocampus, as castration in early puberty increases survival and maturation of granule neurons in the dentate gyrus, while there are no changes in cell proliferation [156]. Changes in spatial reasoning and memory tasks involve alterations in synaptic plasticity, and androgens play a significant role in this regard in the adult hippocampus [21, 157]. There is also evidence that androgens modulate hippocampal synaptic plasticity during adolescence. Male mice undergo loss of dendritic spine synapses in the CA1 hippocampal subregion over the course of puberty, an effect largely reversed by gonadectomy [158], although prepubertal gonadectomy does not prevent a similar loss of synapses in female rats [159]. Functionally, adult male rats have reduced social memory compared to juveniles, coinciding with a shift from long-term potentiation to depression in response to stimulation in the CA1 region of the hippocampus. Both gonadectomy and androgen receptor antagonism at the beginning of puberty, but not later in adolescence, prevent the developmental shift to long-term depression and improve social memory in adults [160]. While these data suggest an organizational role for pubertal androgens in males, whether these are truly sexually differentiated responses is not clear, as comparative treatments using both males and females were not included in these studies. Interestingly, these cellular effects of androgen signaling in the adolescent hippocampus are opposite those in the adult, where androgens promote cell proliferation and survival in the dentate gyrus [19], and synaptogenesis in Ammon’s horn [21, 161].

## Perspective and significance

Although the data are not nearly as extensive as what is known in adults, the influences of testosterone and its metabolites on the hippocampus during development are seen at the biochemical, morphological, and functional levels, and a few general conclusions can be drawn. First, rodent models demonstrate that males and females differ in the strategies they use during hippocampal-dependent learning, which is reflected in differential engagement of the hippocampus as a function of sex. This sex difference in hippocampal dependency is programmed during neonatal development, and for some parameters requires the aromatization of testosterone to estradiol. The effects of neonatal estradiol signaling on the hippocampal circuitry include alterations in cell genesis and microglia activity that may have long-term consequences for the content of various cell types within the hippocampus. The direct effects of androgen signaling in the neonatal hippocampus largely involve positive effects on dendritic arborization and synaptic connectivity, and these too persist into later life. Changes in hippocampal cytoarchitecture and circuitry directed by steroid signaling during the perinatal period must be due to altered biochemical changes with the cell, and these are evident in altered calcium influx and downstream activation of transcription factors in response to amino acid neurotransmitters. Ultimately, these will drive steroid-hormone-dependent changes in gene expression. While there is little information concerning gene expression in the developing hippocampus in response to sexual differentiation or steroid hormone signaling [87, 162], robust and high-throughput methodologies in transcriptome analysis can be put to good use in filling this gap. Characterization of transcriptional changes in the hippocampus during the sensitive early developmental period can aid in identifying points of therapeutic intervention in the context of early-life risk factors that promote many neurological and neurodevelopmental disorders, such as trauma, hypoxia/ischemia, and inflammation.

During adolescence, a primary driver of hippocampal maturation appears to be the rise in testosterone during puberty. Androgen-mediated effects in the hippocampus during this period include synaptic pruning and shrinking of dendritic arbors, and these are associated with a shift toward synaptic depression in males as gonadal steroids rise, and decreased social memory. This may have implications for understanding the association between elevated androgens in males and females with ASD [163, 164], and improvements in sociability reported for adolescents treated clinically with anti-androgenic therapies [165]. The available data in humans indicate that testosterone, and not ovarian steroids, are responsible for puberty-driven changes in hippocampal growth and function, although the aromatization of circulating testosterone in the adolescent brain is certainly a possibility. In addition, with the exception of sex differences in cellular proliferation, there is scant evidence that sex chromosomes play a significant role in long-term hippocampal development. This is supported by the clinical data concerning aneuploid adolescent males, but also is indicated in a study using the Four Core Genotype (4CG) mouse model. In the 4CG mouse, the *Sry* gene, which is encoded on the Y chromosome and directs testicular development, is translocated to an autosome, where it directs normal testicular development outside the context of the Y chromosome. This enables the generation of both XX and XY animals that can be male or female according to their gonadal status [166]. On tests of hippocampal-dependent spatial learning, XX males perform as well as XY males, and both XX and XY males perform better than XX and XY females, indicating that the presence of testosterone during development, but not the X or Y chromosome, is important for modulation of hippocampal function [167]. Generally, what emerges from the clinical data and studies in animal models is the primacy of developmental testosterone exposure, during the perinatal and pubertal periods, in both programming and modulating hippocampal function. Understanding the processes by which this occurs will not only elucidate steroid hormone-dependent mechanisms of brain development generally, but may also help identify ways to mitigate the burden of the many neurodevelopmental disorders that involve hippocampal function.

## Data Availability

Not applicable.
